# Development of pH Sensitive Nanoparticles for Intestinal Drug Delivery Using Chemically Modified Guar Gum Co-Polymer

**Published:** 2016

**Authors:** Vegesna Naga Sravan Kumar Varma, Hosakote Gurumalappa Shivakumar, Veerna Balamuralidhara, Manne Navya, Umme Hani

**Affiliations:** *Department of Pharmaceutics, JSS College of Pharmacy, JSS University, Mysore, Karnataka-570015, India*

**Keywords:** pH sensitive nanoparticles, PAAm-g-GG, intestinal drug delivery, guar gum, Esomeprazole

## Abstract

The aim of the research work was to chemically modify guargum (GG) as a pH sensitive co-polymer and formulating intestinal targeting ESO nanoparticles (NPs) using the synthesized co-polymer. Poly acrylamide-grafted-guar gum (PAAm-g-GG) co-polymer was synthesized by free radical polymerization. Chemical modification of PAAm-g-GG by alkaline hydrolysis results in formation of a pH-sensitive co-polymer. The effect of GG and acryl amide (AAm) on grafting was studied. Esomeprazole magnesium (ESO) loaded pH sensitive NPs were prepared by nano-emulsification polymer crosslinking method and characterized. Sixteen formulations were prepared and the concentration of process variables wasvaried to obtain nanoparticles of 200-600 nm. The NPs were found to be homogenous in size distribution. The encapsulation efficiency and drug loading ranged from 33.2% to 50.1% and 12.2% to 17.2% respectively. Particle size, encapsulation efficiency and drug loading increasedalong with co-polymer concentration. *In-vitro* release studies at pH 1.2 for 2 h, followed by pH 6.8 showed that environment pH significantly affected the drug release. SEM has shown that NPsare spherical with smooth surface. The pH sensitive PAAm-g-GGNPs resisted the initial release of the drug from the drug loaded NPs in acidic pH and delayed the release process to a longer period in alkaline environment.

## Introduction

Targeted delivery to intestinal segments mainly colon and duodenum is highly desirable for the local treatment of bowel diseases like ulcerative colitis, crohn’s disease, colonic cancer, duodenal ulcers and for systemic delivery of proteins and peptide drugs (1). The major categories of drugs that are employed for intestinal targeting include antiulcer, antibiotic, anticancer, protein and peptide drugs. The stability of most of these drugs decreases in acidic medium. It has proven to be a challenge to achieve adequate and consistent bioavailability levels for these orally-administered drugs (2). Hence the primary focus of any intestinal targeted delivery system is protecting the drug in its route to intestine (*i.e*. drug release and absorption should not occur in the stomach and bioactive agent should not be degraded) and to allow drug release only in the colon or duodenum (3). Various approaches and dosage forms have been proposed in the past years to serve this purpose to the best.

Nano-sized colloidal carriers composed of natural or synthetic polymers have also been investigated for intestinal targeting of various drugs and peptide molecules. Indeed, it has been demonstrated in experimental colitis that nano-sized particles are taken up more readily by immune related cells such as macrophages or dendritic cells at the area of active inﬂammation, which helps in locally delivering higher amounts of entrapped drugs (4). In recent years, considerable attention has been focused on use of natural hydrophilic polysaccharides for preparation of nano-particles, because of their ﬂexibility to obtain a desirable drug release proﬁle, cost-effectiveness and broad regulatory acceptance. Among the hydrophilic polysaccharides, guar gum (GG) has been reported as a potential carrier for intestinal targeting in pure as well as modified forms (5-8). To further enhance its efficiency of delivering drug specifically to intestine, guar gum can be modified chemically as a pH sensitive polymer.

Chemical grafting is one of the most effective methods of modifying structure and properties of biopolymers (9). Poly (acrylic acid) (PAA) and its derivatives have been reported to be pH-responsive polyelectrolytes, which have been widely used for drug delivery to speciﬁc regions of the gastrointestinal tract (10). The introduction of polyelectrolyte functional group renders the PAAm-g-GG matrix into a poly-anionic polysaccharide network and the weakly ionic functional group on the polymeric chain will make them pH-responsive (11). Based on these previous researches, the possibilities of formulating pH sensitive nanoparticles (NPs) using a chemically synthesized pH sensitive guar gum derivative has been explored. Esomeprazole magnesium (ESO) which is a proton pump inhibitor was used as the model drug. ESO is effective in the treatment of duodenum ulcer. In both *H. pylori *infection and *Zollinger Ellison* syndrome one need to administer drug for a longer period of time. ESO undergoes ionization in gastric pH as such, hence it is not absorbed in stomach. Delivering of drug locally in the duodenum and prolonging the residence time can be a very good strategy to improve the efficacy, which can allow more of the drug to diffuse through the duodenal mucus layer. Hence, sustained release formulations like mucoadhesive pH sensitive NPs prepared by using natural gums are preferred. This leads to the continuous release of drug in duodenum mucosa.

In the present work, an attempt has been made to chemically modify the biopolymer, GG by free radical polymerization and hydrolysis reactions to synthesize a pH responsive co-polymer. NPs of ESO was formulated using the synthesized co-polymer to prevent the degradation, release of drug in acidic conditions and specifically release the drug in alkaline conditions, thereby introducing a new approach in the stream of environmentally responsive NPs for intestinal targeting ‘using guar gum’.

## Experimental

Esomeprazole magnesium was obtained as a gift sample from Dr.Reddy's Laboratories Ltd, Hyderabad, India. Guar gum, acryl amide, ammonium per-sulphate, glutaraldehyde, span 80 and glycerol were purchased from LOBA ChemiePvt Ltd, Mumbai, India. All the other reagents were of analytical grade.

*Preparation of Poly acrylamide-grafted-guar gum (PAAm-g-GG) co-polymer*

*Synthesis of PAAm-g-GG: *The co-polymer PAAm-g-GG was synthesized by free radical polymerization (11, 12). To study the effect of grafting efficacy the amount of GG and AAm is varied. Briefly, 0.5-2.5 g of GG dissolved in distilled water andhydrated for 4 h. The flask was heated at 80˚C followed by addition of 0.5-1.2 g of AAm and 0.5 g of ammonium per-sulphate (APS). Polymerization was carried out for 1 h. The resulting co-polymer was cooled at ambient temperature. 

*Separation of homopolymer* *: *The product was poured in excess methanol and kept for 24 h. The co-polymer was then filtered, washed repeatedly with methanol, dried at 50˚C over night to obtain *PAAm-g-GG*.

*Alkaline hydrolysis of PAAm-g-GG*: Two grams of PAAm-*g*-GG co-polymer was dissolved in 100 mL of 0.9M NaOH solution and stirred at 75˚C for 60 min in a thermostatic water bath, cooled and poured into excess of methanol. The hydrolyzed co-polymer was separated by filtration and washed repeatedly with methanol and dried overnight at 50˚C. The prepared co-polymer was characterized by FT-IR studies.

*Determination of the grafting parameters*

The grafting parameters; percentage grafting ratio (%G), percentage grafting efficiency (%E) and percentage homopolymer (% H) werecalculated according to Fanta’s definitions (13).

Equation 1 $$\mathrm{\%G}=\frac{\mathrm{weight of grafted polymer}}{\mathrm{weight of substrate}}\times 100$$

Equation 2 $$\%E=\frac{\mathrm{weight of polymer in graft}}{\mathrm{weight of polymer formed}}\times 100$$

%H=100-%E

Equation 3

$\mathrm{Rate of grafting},Rg=\frac{\mathrm{weight of grafted polymer}}{\mathrm{volume}\times \mathrm{time}\times \mathrm{moleculat weight of acrylamide}}$

Equation 4

*Effects of the variables on the grafting parameters*

The effect of two major variables, AAm and GG, on the grafting parameters was studied by determining the grafting parameters at various concentrations of the variables. Based on the results the concentrations of acrylamide and GG to be taken were optimized.

*Preparation of PAAm-g-GG nanoparticles*

ESO loaded pH sensitive NPs were prepared by Nano emulsification polymer crosslinking method (14, 15). 100mg of ESO was dissolved in 10 mL of chloroform, to form an oil phase. To this solution, span 80 was added under stirring, which was then added to aqueous guar gum solution under constant magnetic stirring. After mutual saturation of the oil and the continuous phase, the mixture was rapidly stirred at very high rpm using Homogenizer model Polytron® PT 1600E (kinematica, Switzerland). Glycerol (stabilizer) was then added, followed by addition of 25% glutaraldehyde solution (cross-linker). Nano-suspension was kept overnight undisturbed. NPs were obtained after centrifugation at 20,000 rpm for 30 min. They were washed with 15 mL Millipore™ water and re-centrifuged. The yielded NPs were lyophilized, harvested in micro centrifuge tubes and preserved in vacuum desiccators. Lyophilization was carried out in freeze dyer (Ilshin lab co, Mumbai)

*Study of process variables*

Three trials have been carried out to study the effect of different process variables, namely concentrations of co-polymer, cross-linker, emulsifier and stabilizer to get NPs in the desired size range of 200–600 nm.

Initially nine formulations F1 to F9 NPS (first trail) were formulated, by varying two different parameters, *i.e*. concentration of PAAm-g-GG (0.5, 1.0 and 1.5 %w/v) and glutaraldehyde concentration (2, 4 and 6 %w/w).Concentrations of other parameters such as emulsifier (span 80) and stabilizer (glycerol) were kept constant. The concentrations of co-polymer and glutaraldehyde were determined based on the particle size results. 

To study the effect of Span80, F10 to F13NPs (second trial) were prepared using 2, 4, 6 and 8% w/w Span 80. 0.5% w/v of PAAm-*g*-GG and 4% w/w glutaraldehyde, which were selected on the basis of the previous study, were used on this trail.

Similarly, to study the effect of stabilizing agent, F14 to F16 NPs (third trail) were formulated by using various concentrations of co-polymer, glutaraldehyde, span 80 (4%) from the first two trails and varying concentrations of glycerol (5, 10, 15 mL). The formulation chart is given in Table 1.

**Table 1:** Formulation chart of ESO loaded PAAm-*g*-GG nanoparticles

**Formulation**	**PAAm-** ***g*** **-GG % w/v /80mL**	**Oil (mL)**	**Span 80 %w/w**	**Glycerol (mL)**	**Cross-linking agent % w/w**
F1	0.5	10	4	10	2
F2	1	10	4	10	2
F3	1.5	10	4	10	2
F4	0.5	10	4	10	4
F5	1	10	4	10	4
F6	1.5	10	4	10	4
F7	0.5	10	4	10	6
F8	1	10	4	10	6
F9	1.5	10	4	10	6
F10	0.5	10	2	10	4
F11	0.5	10	4	10	4
F12	0.5	10	6	10	4
F13	0.5	10	8	10	4
F14	0.5	10	4	5	4
F15	0.5	10	4	10	4
F16	0.5	10	4	15	4

*Fourier transform infrared (FT-IR) spectroscopy*

The gums, pure drug and the formulations were subjected to FT-IR analysis by KBr pellet method using Fourier-Transform Infrared spectrophotometer, (Shimadzu, FT-IR 8400S, Japan). 

*Differential scanning calorimetry (DSC)*

Differential scanning calorimetric studies were carried out for pure drug and formulations using differential scanning calorimeter (Shimadzu corporation, DSC 60, Japan). The instrument was calibrated using high purity indium metal as standard. The dynamic scans were taken in nitrogen atmosphere at a heating rate of 10˚C min^-1^.

*Determination of particle size, zeta potential and PDI*

The particle size, zeta potential and PDI of prepared formulations were characterized using Malvern zetasizer (DTS Ver.5.10, Serial No. MAL1031371, Malvern Instruments Ltd, UK.). The experiment was performed using clear disposable zeta cell, water as a dispersant which has refractive index (RI)-1.330 and viscosity (cP)-0.73 and the temperature was kept constant at 25˚C.

*SEM studies*

The surface morphology of samples was determined using scanning electron microscope (SEM). The samples were fixed on SEM sample holder with a double sided adhesive tape and coated with a layer of gold of 150A for 2 minusingsputter coater in a vacuum of 3x10^-1^atm of argon gas. The sample was then examined using a scanning electron microscope (JSM-840 A scanning microscopy, Tokyo, Japan).

*Encapsulation efficiency of NPs*

Entrapped drug within the NPs was estimated by subjecting the nano-particle dispersion to centrifugation at 5,000 rpm for 30 min. The supernatant containing un-entrapped drug was removed (16, 17). The sediment of NPs was washed again with buffer to remove any un-entrapped drug and the washings were combined with supernatant for spectrophotometric analysis at 301 nm.

Encapsulation efficiency was calculated using formula:

$\mathrm{Encapsulationefficiency \%}=\frac{\mathrm{Total drug}-\mathrm{Unentrapped drug}}{\mathrm{Total drug}}\mathrm{x100}$

Equation 5

*Determination of drug loading*

A weighed amount (50 mg) of NPs was suspended in small amount of methanol and sonicated for 15 min in order to extract the entrapped drug. The solution was filtered through Whatman™ filter paper and the filtrate was analyzed spectrophotometrically at 301 nm after suitable dilutions with pH 6.8 phosphate buffer. 

Drug content was calculated as:

Equation 6 Drugcontent=Conc from standard graph x dilution factor1000

*In-vitro drug release studies (* 18 *)*

NPs containing 50 mg of drug were packed in a dialysis bag (MW cut-off 3500) and incubated in 50 mL of simulated fluids at 37 ± 0.5˚C with slow magnetic stirring. To simulate the environment of upper gastro-intestinal tract, the release behavior of ESO was also investigated in variable pH conditions, in which the NPs were first incubated in 50 mL of simulated gastric fluid (pH 1.2) for 2 h, and then transferred into 50 mL of pH 6.8 buffer. At specific time intervals samples were withdrawn and replaced with equivalent amount of same buffer. The samples were filtered and analyzed using UV spectrometer at 301 nm.

To determine the mechanism of drug release from the films, the release data was fitted to zero-order, first-order, and Higuchi models using the PCP DissoV-2.08 software. 

## Results and Discussion

*Characterization of PAAm-g-GG*

**Figure 1:**
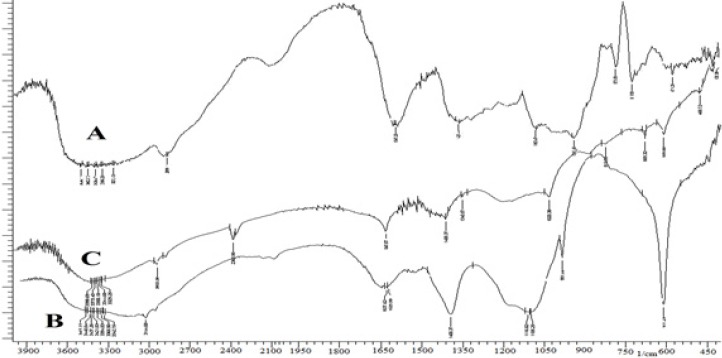
FT-IR spectra of GG (A), PAAm-g-GG (B) and hydrolyzed PAAm-g GG (C).

The grafting efficiency was found to be 83.56%. FT-IR spectra of GG, PAAm-*g*-GG and physically hydrolyzed PAAm-g-GG are given in Figure 1.The pure GG showed a broad peak at 3,415 cm^-1^ due to the presence of hydrogen bonded OH groups. In the spectrum of PAAm-g-GG, additional peaks were observed at 3,438, 3,144, 1,637 and 1,400 cm^-1^. The peak at 1,105 cm^-1^ was due to the presence of the ether linkage formed by the reaction between OH groups of GG and acrylamide. The peak appearing at 3,144 cm^-1 ^was absent in the spectrum of alkaline hydrolyzed PAAm-g-GG indicating the absence of N-H band. The peaks at 1,617 and 1,400 cm^-1^ are due to COO- groups. Thus, during the alkaline hydrolysis-CONH_2_ groups of PAAm present on the back bone of GG are converted to –COOH functional groups resulting in pH sensitive polymer.

**Figure 2:**
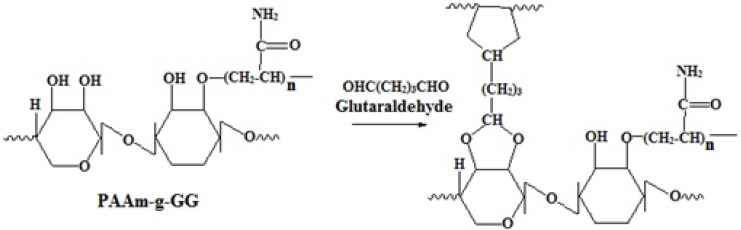
Schematic representation of Graft polymerization of acryl amide on guar gum

The grafting of PAAm on the backbone of GG was carried out by free radical polymerization. The free radical formed here was the alkoxy radical of GG. APS which acts as reaction initiator under goes decomposition at a temperature of 80˚C to produce sulfate anion free radical, which abstracts the hydrogen from the hydroxyl groupat position two of GG to form alkoxy radical on the substrate. Then the resulting macro radical initiates the graft polymerization of AAm on to the backbone of GG by an ether linkage. The reaction is illustrated in Figure 2.

*Effect of acrylamide on grafting parameters*

When the polymerization reaction was carried out at different concentrations of acrylamide, it was observed that with the increase in concentration of acrylamide up to 0.8 g, %G, %E and R_g _increased but %H decreased (Table 2). Beyond this concentration %G, %E and R_g _decreased. The increase in grafting parameters was due to a greater addition of acrylamide molecules to the growing grafted chains. The greater availability of acrylamide resulted in chain initiation. Thereafter, it became a free-radical donor to the neighbouring molecules. Thus it leads to formation of many grafted chains. 

**Table 2:** Effect of acrylamide concentration on grafting parameters

**Acrylamide(g)**	**%G**	**%E**	**%H**	**R**_g_
0.4	86.2	37.6	62.4	14.3
0.6	102.4	56.8	43.2	22.6
0.8	123.2	68.1	31.9	34.4
1.0	76.7	31.3	68.7	23.6
1.2	42.5	18.9	77.1	9.3

Addition of acrylamide beyond 0.8 g increased the viscosity of the reaction medium due to the formation of poly acrylamide. The primary free radicals that were formed transferred the electron to the monomer, which produced more homopolymer. This could be the reason for increase in %Hat higher concentrations of acrylamide.

*Effect of guar gum on grafting parameters*

Table 3 reveals that with increase in concentration of guar gum from 0.5 to 2 g, %G and R_g _increased. Beyond this concentration,further increase of %G and R_g_ was not observed. However, the %E was found to enhance along with acrylamide. The homopolymer concentration decreased continuously.

**Table 3:** Effect of guar gum concentration on grafting parameters

**Guar gum (g)**	**%G**	**%E**	**%H**	**R**_g_
0.5	71.8	23.6	76.4	11.5
1.0	85.3	37.2	62.8	19.4
1.5	94.2	53.7	46.3	26.7
2.0	123.2	68.1	31.9	34.4
2.5	53.5	75.3	24.7	6.7

The increase in %G and R_g _up to a certain extent was due to the availability of more grafting sites, which were produced by the reaction of the guar gum molecule with ACM. At higher concentration above 2 g, the increased viscosity of the medium and giant size of the molecules decreased the availability of the free radicals.

*Preparation of drug loaded PAAm-g-GG nanoparticles*

**Figure 3:**
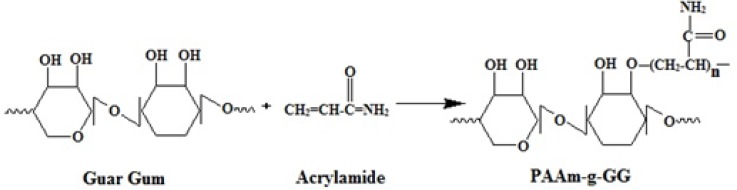
Schematic representation of Crosslinking of PAAm-g-GG copolymer with glutaraldehyde.

For the formulation of nanoparticles, PAAm-g-GG was used as co-polymer, chloroform as a solvent, span 80 as an emulsifier and antifoaming agent, glycerol as stabilizer and glutaraldehyde as crosslinking agent. Span 80 helps in transfer of ESO to PAAm-g-GG during controlled evaporation of the solvent. The low HLB value of span 80 helps in transfer of ESO to PAAm-g-GG rather than water. Higher HLB value of the emulsifier will hinder detachment of ESO from the solvent and transfer to PAAm-g-GG. Hence span 80 is producing the right balance in changing weak interactive forces in the process. Glutaraldehyde causes cross linking with the active primary hydroxylic groups of the galactose and mannose unit of PAAm-g-GG by H-bonding, interfering with the presence of water to the hydroxyl group of PAAm-g-GG (15). The crosslinking reaction is represented in Figure 3.

*Characterization of PAAm-g-GG nanoparticles*

*FT-IR studies*

**Figure 4:**
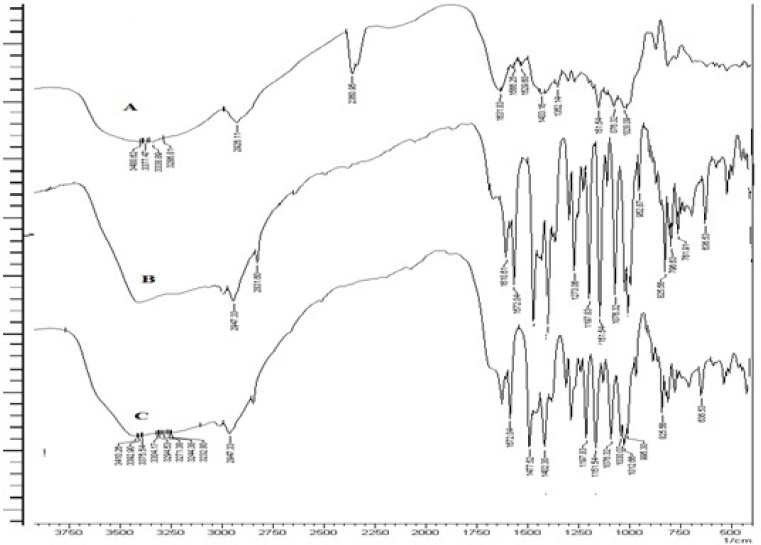
FT-IR spectra of GG (A), PAAm-g-GG (B) and hydrolyzed PAAm-g GG(C

The obtained spectra of ESO and formulation are reported in Figure 4. It was observed that there was no disappearance or significant shift in the position of major peaks that corresponded to various groups of ESO in any spectra of formulation. This proves that the grafted guargum copolymer is compatible with the drug.

*Differential scanning calorimetry (DSC)*

**Figure 5:**
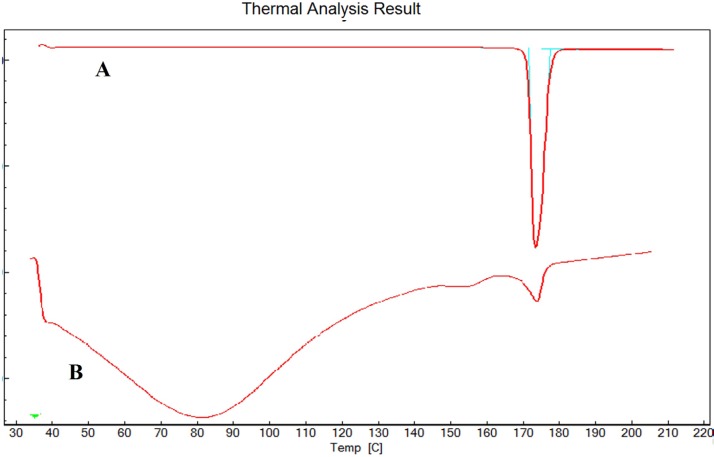
DSC of ESO (A) and F8 (B).

 As reported in Figure 5, the DSC thermogram of pure ESO and formulation F8 showed an endothermic peak at 174˚C and 172.7˚C respectively which corresponds to the melting point of drug. No significant change in the melting point of drug indicates absence of any interactions between the drug and synthesized co-polymer.

*Particle size, zeta potential and PDI analysis*

The results of particle size, zeta potential and PDI of formulations (F1-F16) are given in Table 4. The particle size was taken as the major criteria for optimizing the concentrations of various process variables in the trails. Based on the results obtained from all the three studies, 10 formulations (F1, F4, F5, F7, F8, F11, F12, F13, F15, F16) which had particle size below 600 nm were selected for the further studies of encapsulation efficiency and drug loading.

**Table 4:** Particle size, zeta potential and PDI of F1-F16

**Formulation**	**Particle size mean ± SD** *	**PDI**	**Zeta Potential (mV)**
F1	572 ± 0.56	0.217	-21.3
F2	945 ± 0.16	0..804	-35.5
F3	1473 ± 0.58	0.843	-36.4
F4	356 ± 0.23	0.109	-25.7
F5	525 ± 0.53	0.693	-19.4
F6	1376 ± 0.28	0.745	-34.3
F7	313 ± 0.74	0.368	-45.6
F8	583 ± 0.32	0.457	-13.8
F9	1258 ± 0.54	0.972	-26.6
F10	1293 ± 0.45	0.854	-17.3
F11	387 ± 0.12	0.424	-18.5
F12	344 ± 0.32	0.365	-29.4
F13	275 ±0.65	0.256	-31.2
F14	1184 ± 0.32	0.834	-14.3
F15	352 ± 0.25	0.376	-23.4
F16	387 ± 0.27	0.492	-27.3

* S.D, n = 3

The particle size results indicated that all the process variables have direct effect on particle size. Increasein co-polymer concentration from 0.5 to 1.5% w/v increased particle size and increasein thecross-linking agent from 2 to 4% w/w decreased theparticle size. As observed from the results of F1 to F9, formulations containing 0.5% w/v concentration of PAAm-g-GG gave the best particle size range. The cross-linker concentration had no effect on the particle size when more than 4% w/w was employed. Hence PAAm-g-GG concentration of 0.5% w/v and cross-linker concentration of 4% w/w were found to be optimal concentrations that can be used. In case of F10 to F13, mean diameter of NPs were found to decrease, with varying concentrations of span 80 from 2% to 8% w/w. Hence 4% concentration of span80 was found to be optimal. In the case of F14 to F16, mean diameter of NPs were found to decrease, with varying amount of stabilizer from 5 to 10 mL and when more the 10 mL stabilizer is used no much significant decrease/increase in particle have been observed. Hence 10 mL glycerol was found to me optimal below these optimum concentration NPs tend to fuse or produced larger size particles. Hence to conclude0.5% w/v of PAAm-*g*-GG, 4% w/w glutaraldehyde, 4% span 80 and 10mL of glycerol were found to be the optimal concentrations for obtaining NPs in size range of 200-600 nm.

In general PDI values ranges from 0.05 to 1. A PDI value of 1 indicates that the sample has a very broad size distribution and may contain large particles or aggregates that could be slowly sediment. Therefore, it can be stated that the prepared NPs were characterized by a homogeneous size distribution. The negative zeta potential of the NPs was due to the presence of carboxylic groups in the co-polymer and the use of non-ionic stabilizer, glycerol.

*Encapsulation efficiency and drug loading of NPs*

The results obtained after determining the encapsulation efficiency and drug loading are given in Table 5. The encapsulation efficiency was found to be 33.2% to 50.1% and percentage drug loading was in the range of 12.2% to 17.2% for the prepared formulations. The encapsulation efficiency and drug loading were found to increase with increase in the co-polymer concentration. Based on the results obtained, 6 formulations (F1, F4, F5, F8, F15 and F16) which had encapsulation efficiency greater than 40% were selected for the *in-vitro* drug release studies.

**Table 5:** Encapsulation efficiency and drug loading of different formulations

**Formulation**	**Encapsulation efficiency % mean ± SD** *	**Drug loading%** **mean ± SD** *
F1	48.32 ± 0.32	13.23 ±0.43
F4	43.43 ±0.45	16.32 ±0.34
F5	50.12 ±0.73	14.23 ±0.28
F7	38.87 ±0.47	15.34 ±0.83
F8	46.34 ±0.36	17.23 ±0.13
F11	39.87 ±0.28	16.32 ±0.35
F12	38.15 ±0.64	12.27 ±0.13
F13	33.21 ±0.21	15.23 ±0.27
F15	40.76 ±0.34	16.74 ±0.38
F16	41.34 ±0.43	15.32 ±0.28

* S.D, n=3

*In-vitro drug release studies*

**Figure 6:**
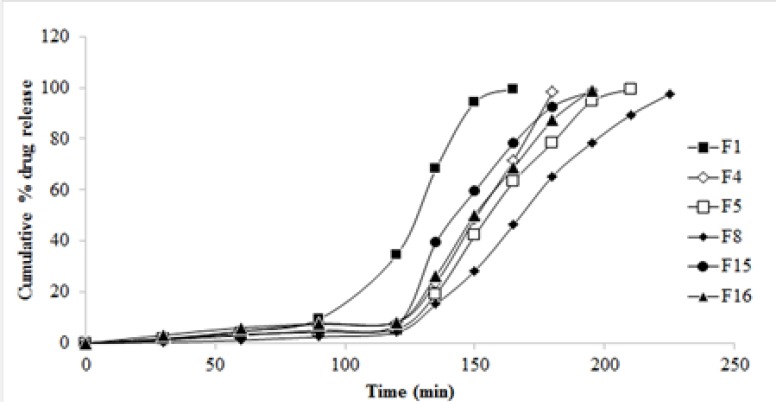
*In-vitro* drug release profile of various formulations

The cumulative drug release profile of selected 6 formulations is given in Figure 6. A significant decrease in the drug release is due to the increase in co-polymer to drug ratio. An increase in guar gum concentration from 0.5 to 1.0% w/v reduced the release of ESO from the PAAm-g-GGNPs. Also with an increase in glutaraldehyde concentration from 2% to 6% w/w decreased cumulative drug release from 99.32 to 46.43% was observed. In the first 2 h, about 4% to 8% of the ESO entrapped within the NPs was gradually released. As the pH changed from 1.2 to 6.8 it was observed that NPs dissolved quickly, achieving 99.83% of the release amount of the loaded ESO. This indicates that environmental pH significantly affected the release of the drug from pH sensitive PAAm-g-GGNPs.

The drug release behavior of the NPs might be influenced by the pH as well as concentrations of co-polymer and cross-linker employed. Various ratios of the co-polymer, glutaraldehyde were tested to optimize the fabrication procedure of NPs.

*Effect of cross-linker on release profile of drug:*

The crosslinking effect of glutaraldehyde significantly reduced the extent of swelling of guar gum by hindering the penetration of the solvent into the NPs as a result of which the release of the drug from the NPs was retarded. It is reported that the swelling rate of polymer is determined by the ability of its water uptake which depends upon the extent of hydrodynamic free volume and the availability of hydrophilic functional groups available to establish hydrogen bonds (12). So, the higher release of drug at lower concentrations of cross linkers was because the polymer matrix had high hydrodynamic free volume to accommodate more solvent molecules, thereby resulting in swelling of polymer matrix.

*Effect of* *co-polymer* *and pH on the drug release profile*:

As it is evident from the results, an increase in co-polymer concentration retards the drug release from PAAm-g-GG NPs which in turn is influenced by pH of the environment. The pH shows its effect on the drug release by influencing the ionization and swelling of the co-polymer. The–COOH functional groups of the synthesized PAAm-g-GG copolymer remains unionized at gastric pH leading to negligible swelling and drug release, but they undergo ionization at higher pH leading to maximum swelling and drug release in intestine. In alkaline pH of intestine the crosslinking of NPs break and water penetrates into the polymer matrix and the polymeric NPs starts swelling. Due to the swelling action, the drug which is dispersed in the polymer matrix begins to diffuse out (12). Thus the release of the drug from the polymer matrix in the present study depends upon two phenomena: ionization in response to environmental pH and the rate of swelling of the polymer matrix. The continued swelling of the matrix causes the drug to diffuse out in alkaline medium. 

Formulation F8 which could extend the release of drug up to 3.75 h was considered to be the best formulation and further studied for surface morphology using SEM. The best fit model of F8 was found to be Korsmeyer-Peppas model. The release exponent n value of 2.934 indicated that the transport of the drug was by super case II transport (drug release by relaxation of polymer and polymer erosion).

*SEM studies*

**Figure 7:**
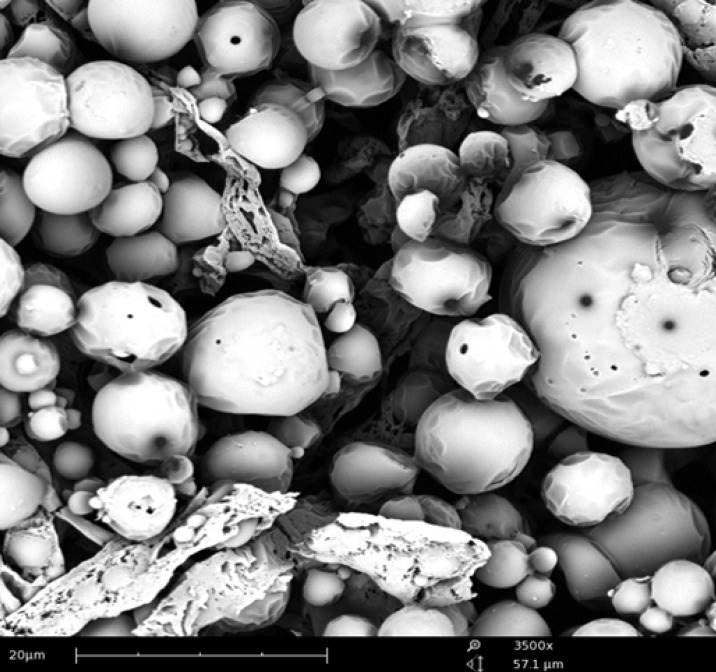
SEM image of F8

SEM image of formulation F8 has shown spherical particles with smooth surface, as given in Figure 7. NPs did not have any porous surfaces or internal porous structure, suggesting that there could be no chance of release of loaded drug by matrix rupture process.

## Conclusion

The results of the study indicate that PAAm-g-GG co-polymer can be successfully synthesized by free radical polymerization of GG with polyacrylamide which on alkaline hydrolysis results in a pH sensitive co-polymer. 0.5% w/v of PAAm-*g*-GG, 4% w/w glutaraldehyde and 4% span 80 could produce Nanoparticles in size range of 200-600 nm. 

When compared to unit oral dosage forms, NPs enabled the drug to reach the intestine quickly and retain for long period of time. Because of their smaller particle size these systems uniformly disperse in the GI tract and also ensure more uniform drug absorption. The pH sensitive PAAm-g-GGNPs resisted the initial release of the drug from the drug loaded NPs in acidic pH and delayed the release process to a longer period in alkaline environment making the present drug delivery system for the antiulcer drug ESO as a sustained drug release system. 

## References

[1] Philip AK, Betty Philip (2010). Colon targeted drug delivery systems: A review on primary and novel approaches. Oman Med J.

[2] Weiwei G, Juliana C, Omid CF (2010). pH-responsive nanoparticles for drug delivery. Mol Pharmaceutics.

[3] Gaurav T, Ruchi T, Pranay W, Ankita W, Awani KR (2010). Primary and novel approaches for colon targeted drug delivery -A review. Int J Drug Del.

[4] Regis C, Laurence P, Vincent P, Christine J, David JB, Yves-Jacques S, Schneider, Veronique P (2013). Drug Delivery to inﬂamed colon by nanoparticles: Comparison of different strategies. Int J Pharm.

[5] Raj Kumar S, Akanksha T (2012). Carbohydrate polymers: Applications and recent advances in delivering drugs to the colon. Carbohydr Polym.

[6] Rema SS, Swapankumar G, Emilia TA (2010). Preparation and characterization of guar gum nanoparticles. Int J Biol Macromol.

[7] Jayanta KS, Rita M, SaibalKanti B, Ranadeep M, Anindya D, Prasun G, Angshuman B (2012). In-vitro cytotoxicity analysis of tamoxifen citrate loaded cross-linked guar gum nanoparticles on jurkat (human t-cell leukemia) cell line. J Drug Del Ther.

[8] Prabaharan M Prospective of guar gum and its derivatives as controlled drug delivery systems. Int J Biol Macromol.

[9] Abdel-Halima ES, El-Rafieb MH, Salem S, Al-Deyab (2011). Polyacrylamide/guar gum graft copolymer for preparation of silver nanoparticles. Carbohydr Polym.

[10] Vikas R, Parshuram R, Ashok KT, Ram SS, John FK, Charles JK (2011). Modified gums: Approaches and applications in drug delivery. Carbohydr Polym.

[11] Kumaresh SS, Anandrao RK, Tejraj MA (2001). Chemically modified polyacrylamide-g-guar gum-basedcross-linked anionic microgels as pH-sensitive drug delivery systems: preparation and characterization. J Control Rel.

[12] Kumaresh SS, Tejraj MA (2002). Water transport and drug release study from cross-linked polyacrylamide grafted guar gum hydrogel microspheres for the controlled release application. Eur J Pharm Biopharm.

[13] Arti S, Vivek M, Shaiendra KS, Rajesh K (2010). Vanadium (V)/mandelic acid initiated graft copolymerization of acrylamide onto guar gum in an aqueous medium. J Appl Polym Sci.

[14] Jayanta KS, Saibal Kanti B, Ranadeep M, Rita M (2009). Preparation of cross-linked guar gum nanospheres containing tamoxifen citrate by single step emulsion In-situ polymer cross-linking method. J Incl Phenom Macrocycl Chem.

[15] Jayanta KS, Rita M, SaibalKanti B, Ranadeep M, Angshuman B (2011). Controlled release of tamoxifen citrate encapsulated in cross-linked guar gum nanoparticles. Int J Biol Macromol.

[16] Dora CP, Singh SK, Kumar S, Datusalia AK, Deep A (2010). Development and characterization of nanoparticles of glibenclamide by solvent displacement method. Acta Pol Pharm.

[17] Loveymi BD, Jelvehgari M, Milani PZ, Valizadeh H (2012). statistical optimization of oral vancomycin-eudragitrs nanoparticles using response surface methodology. Iran J Pharm Res.

[18] Dharamadhikari NR, Joshi SR, Manekar NC (1991). Preparation and in-vitro evaluation of salbutamol sulphatemicroencapsules. J Microencapsul.

